# Hydrophobic Starch-Based Films Using Potato Washing Slurries and Spent Frying Oil

**DOI:** 10.3390/foods10122897

**Published:** 2021-11-23

**Authors:** Sílvia Petronilho, André Oliveira, M. Rosário Domingues, Fernando M. Nunes, Manuel A. Coimbra, Idalina Gonçalves

**Affiliations:** 1 LAQV-REQUIMTE, Department of Chemistry, Campus Universitário de Santiago, University of Aveiro, 3810-193 Aveiro, Portugal; andreoliveira98@ua.pt (A.O.); mrd@ua.pt (M.R.D.); mac@ua.pt (M.A.C.); 2 Chemistry Research Centre-Vila Real, Department of Chemistry, University of Trás os-Montes and Alto Douro, Quinta de Prados, 5001-801 Vila Real, Portugal; fnunes@utad.pt; 3 Mass Spectrometry Centre, Department of Chemistry, Campus Universitário de Santiago, University of Aveiro, 3810-193 Aveiro, Portugal; 4 CESAM, Centre for Environmental and Marine Studies, Department of Chemistry, Campus Universitário de Santiago, University of Aveiro, 3810-193 Aveiro, Portugal; 5 CICECO—Aveiro Institute of Materials, Department of Materials and Ceramic Engineering, Campus Universitário de Santiago, University of Aveiro, 3810-193 Aveiro, Portugal; idalina@ua.pt

**Keywords:** potato chip byproducts, biobased films, alkaline catalyst, transesterification, single step, hydrophobicity, stretchability, water vapor barrier

## Abstract

Starch is a promising candidate for preparing biodegradable films with useful gas barriers and thermoplastic capabilities. However, these materials are hydrophilic and brittle, thus limiting their application range. To overcome these drawbacks, it has been hypothesized that starch can be hydrophobized and plasticized during the starch-based film production using a single-step approach and following transesterification principles. In this work, KOH powder and spent frying oil (SFO) were used as an alkaline catalyst and a source for triacylglycerides, respectively, to promote the modification of starch. Different ratios of SFO (*w*/*w* related to the dried starch weight) were tested. When compared to the neat films (without a catalyst and SFO), the incorporation of at least 15% SFO/KOH gave rise to transparent, hydrophobic (water contact angles of ca. 90°), stretchable (*ca*. 20×), elastic (*ca*. 5×), and water tolerant starch-based films, contrary to the films produced without the catalyst. ATR-FTIR and ^1^H NMR revealed structural differences among the produced films, suggesting that starch was modified with the SFO-derived fatty acids. Therefore, adding KOH during the potato starch/spent frying oil-based film’s production was determined to be a promising in situ strategy to develop starch-based materials with improved hydrophobicity and flexibility, while valorizing the potato chip industry’s byproducts.

## 1. Introduction

Starch, a carbohydrate composed of linked d-glucose units in the form of amylose and amylopectin [1], stands out as a promising natural polymer for developing biodegradable films due to its film-forming ability with thermoplastic and interesting gas barrier properties [2,3,4], along with its high availableness, reduced cost, and renewability [5]. To avoid the use of edible starch in the material’s development, starch obtained from agri-food industry byproducts has been considered [6,7]. For instance, starch recovered from the potato industry’s washing slurries recently showed physicochemical, thermal, and rheological properties that were competitive with commercially available starch (directly extracted from the potato) and suitable for use in the development of biobased materials [8]. Nevertheless, a shortcoming of starch is its inherent hydrophilic nature [9] due to its hydroxyl-rich structure that can form hydrogen bonds with water, thus it creates materials that have a high water sensitivity and are of a hygroscopic nature [10]. This drawback has limited the application range of starch-based material and has hampering its widespread usage in the biobased and biodegradable plastics industry.

Chemical modification has been studied [5,11,12] for improving the hydrophobicity of starch, which mainly stands on the replacement of the hydroxyl functional groups of the starch’s backbone with huge hydrophobic groups that have no capacity to form hydrogen bonding with water molecules [13]. The direct esterification of starch with free fatty acids [14,15,16] or with fatty acid derivatives, such as chlorides [10,17] and anhydrides [18,19,20], which are thermodynamically favorable, is one of the strategies used. Besides hydrophobization, starch esterification with fatty acids also increases its thermal stability and processability properties [5,11,12]. However, these multi-step approaches are time consuming and normally involve the use of organic solvents or mixtures of solvents to first solubilize the starch before proceeding with its chemical modification [10,17,18,19,20]. Moreover, an enzymatic pre-treatment using triacylglyceride sources (such as vegetable oils) is also normally required to release the fatty acid derivatives for further use in starch esterification [14,15,16]. Transesterification with highly pure soybean oil [21] and analytical grade esters, such as vinyl laurate and stearate [22], both in DMSO and when using alkaline catalysts, has also been applied to modify the hydrophilic nature of commercial starch. Similarly, hydrophobic starch can also be obtained from analytical grade fatty acid methyl esters (FAMEs) using densified carbon dioxide in the presence of potassium carbonate in a high-pressure reactor [23]. When used in paper coating, the transesterified starch was able to provide high hydrophobicity and prolonged water resistance to the cellulose-based materials [21].

To the best of our knowledge, none of the already published works deal with the modification of starch during the formation of biobased films, but have always focused on a two-step reaction procedure for the modification of granular starch followed by its processing as a biomaterial. In this work, it was hypothesized that, in the presence of potassium hydroxide (KOH), starch can be in situ hydrophobized and plasticized during the production of starch/potato chips’ spent frying oil-based films, following transesterification principles. The reaction mechanism involved in the transesterification of starch in the presence of triacylglycerides is presumably similar to the alcohols’ transesterification with triacylglycerides, forming fatty acid esters [24,25]. In this reaction, the alcohol is transformed into an alkoxide by an alkaline catalyst (such as KOH used in this work). After this, the triacylglycerides’ carbonyl group is attacked by the alkoxide, thus forming an alkyl ester and an anion of diacylglyceride that instantaneously captures a proton from the medium. Without the presence of the catalyst, no transesterification reaction occurs between the oil-derived fatty acids and the OH groups of the starch. The conditions that were used needed to be a compromise between promoting the transesterification in the presence of KOH as catalyst and avoiding the saponification reactions that can be promoted by KOH.

Under a circular economy concept, and as a sustainable alternative of channeling foodstuffs for materials purposes, starch and esterified fatty acids were recovered from potato washing slurries and potato chip spent frying oil, respectively, two non-value byproducts of the potato chip industry.

## 2. Materials and Methods

### 2.1. Materials

Potato chip industry byproducts, namely washing slurries and frying residues, were provided by A Saloinha, Lda. (Mafra, Portugal). Starch was recovered from the lyophilized potato washing slurries, which was formed by 25 % amylose and had a gelatinization temperature and enthalpy of 59–71 °C and 12.5 J/g, respectively [8].

Potassium hydroxide (KOH, 99.9% purity) was purchased from José Manuel Gomes dos Santos (Odivelas, Portugal), glycerol was from Scharlab S.L. (Barcelona, Spain), and sodium azide, chloroform, and methanol were from Sigma-Aldrich (Lisboa, Portugal). Analytical grade reagents were used with no further purification.

### 2.2. Recovery and Characterization of Potato Chip Spent Frying Oil

Potato chip spent frying oil (SFO) was recovered from 60 g of potato chip industry frying residues using Soxhlet extraction, at 70 °C, for 5 h (*ca.* 15 extraction cycles) with a chloroform/methanol mixture (2:1 *v*/*v*). Then, the remaining water was removed using anhydrous sodium phosphate, and the solvent was evaporated in a rotary evaporator until the samples’ weight was constant. The recovered SFO yield (g of lipid per 100 g of potato chip frying residues) was determined in three independent replicates. The SFO was stored in dark conditions, at room temperature, until further use.

The fatty acid methyl esters (FAMEs) were determined after the SFO fraction alkaline-catalyzed transesterification [26] using 5 mg of SFO andheptadecanoate methyl ester (1.5 mg/mL, in *n*-hexane) and KOH methanolic solution (2 M) as the internal standard and the catalyst, respectively. The FAMEs were analyzed in a GC-FID Perkin Elmer Clarus 400 equipped with a DB-FFAP column (30 m × 0.32 mm (I.D.) × 0.25 μm film thickness, J&W Scientific Inc., Folsom, CA, USA). The injection was in split mode with 20:1 (5 min) ratio. The GC injection port and the detector were programmed at 245 and 250 °C, respectively. The oven program followed 3 temperature ramps as already described [8]. The identification of the FAME compounds was based on the comparison of their retention times with the ones obtained by a commercial FAME mixture (C_8_–C_24_) injection.

The peroxide value of the recovered SFO was evaluated by titration according to the American Oil Chemists’ Society (AOCS) official methods [27]. Briefly, 5 g SFO was dissolved in 30 mL acetic acid/chloroform mixture (3:2 *v*/*v*) to which saturated potassium iodide solution (0.5 mL) was then added. This mixture was then incubated in the dark (1 min) and diluted with distilled water (30 mL). The titration occurred with a sodium thiosulfate solution (0.01 N) in the presence of a starch suspension (10%, 0.5 mL) until the dark blue color disappeared. A total of 3 independent replicates were performed and the peroxide value was expressed in milliequivalents of peroxide per kg of the oil sample.

### 2.3. Starch-Based Film Production

The starch-based films were produced following a previously described solvent casting method [8]. Briefly, a suspension of potato starch (4% *w*/*v*) containing KOH (10% *w*/*w* related to the SFO weight basis), as alkaline catalyst, glycerol (30% *w*/*w* related to the starch dry weight basis), as a plasticizer, and SFO in different proportions (10%, 15%, and 20% *w*/*w* related to starch dry weight basis). Each dispersion was gelatinized at 95 °C ± 0.1, for a total of 45 min with constant stirring (*ca*. 500 rpm). The obtained gelatinized suspension was then degassed with a vacuum pump, spread onto plexiglass plates, and dried (25 °C, overnight). Films without KOH and SFO were used as controls.

### 2.4. Film Characterization

#### 2.4.1. Wettability

Water contact angles (WCAs) of the developed films’ surfaces were evaluated using a tensiometer (OCA 20, Dataphysics) equipped with an automatic image capture system (Dataphysics SCA20 M4), as described [7]. Briefly, 3 μL ultrapure water were dropped in the film strips’ surfaces (1 cm × 10 cm) and the WCAs were calculated following the Laplace–Young equation [28]. At least 10 droplet images were obtained along each strip length. The measurements were performed on the two surfaces of each produced starch-based film, i.e., the one exposed to air during casting (film upper surface) and the other one which was in contact with the plexiglass plates (film lower surface).

#### 2.4.2. Mechanical Properties

The mechanical properties of each developed starch-based film were determined using texture analyzer apparatus (model TA. Hdi, Stable Micro Systems), as already described [7]. Briefly, each film was cut (10 strips each of size 1 × 9 cm) and the thickness was measured at 4 different points using a digital micrometer (*ca*. 0.001 mm accuracy; Mitutoyo Corporation, Kanagawa, Japan). A total of 6 specimens of each developed film were tested. Tensile strength, elongation at break, and Young’s modulus values were calculated based on the ASTM D 882 83 standard method.

#### 2.4.3. Fourier-Transform Infrared (FTIR) Spectroscopy Analysis

The FTIR analysis of the films was determined by a Golden Gate single reflection diamond ATR system in a Perkin Elmer Spectrum BX spectrometer (Perkin Elmer, Inc., Hopkinton, MA, USA). FTIR spectra were taken at a resolution of 16 cm^−1^ and with double bi-directional scans, 32 co-added scans, and a wave number range between 4000 and 500 cm^−1^ (mid infrared region). The FTIR analyses were performed in five replicates of the film squares (4 cm^2^) that had been previously washed in *n*-hexane. To allow a comparison among the different film samples under study each spectrum was normalized.

#### 2.4.4. Proton Nuclear Magnetic Resonance Spectroscopy (^1^H NMR) Analysis

The NMR measurements were conducted to obtain detailed information on the structure of the developed films. The ^1^H NMR spectra were acquired on a Bruker Advance III TM HD 500 MHz liquid-state NMR spectrometer (narrow bore), 11.75 T magnetic field (ultrashield ascend). Prior to the analysis, each film sample was dissolved in dimethyl sulfoxide (DMSO)-d6 at room temperature. The experiments were carried out at 10 °C with a double resonance 5 mm broadband observation probe with inverse capabilities and N_2_ cryocontrol unit for prodigy probe with z-gradients. All chemical shifts were in parts per million (ppm). The measurements were performed in triplicate, each one corresponding to an independent experiment.

#### 2.4.5. Chromatic Properties

The chromatic properties of each film were evaluated using tristimulus colorimetry (CIELab). The L* (luminosity), a* (red/green color), and b* (yellow/blue color) chromatic coordinates were analyzed using a CR-400 Chroma Meter [29]. The films’ total color difference (∆E) was calculated using Equation (1):

(1) $$\Delta E=\surd {\left(\Delta L^{*}\right)}^{2}+{\left(\Delta a^{*}\right)}^{2}+{\left(\Delta b^{*}\right)}^{2}$$

where ΔL*, Δa*, and Δb* are the L*, a*, and b* differences among films with KOH and SFO and the neat starch-based films.

#### 2.4.6. Water Solubility

Each film square (4 cm^2^) was weighed, immersed in an aqueous solution (30 mL) containing 0.02% (*v*/*v*) of sodium azide, and kept with orbital agitation (80 rpm, room temperature, 7 days). The films were then dried (105 °C, overnight), cooled down to room temperature, and weighed [7]. The weight loss percentage was determined to evaluate the solubility of each film (Equation (2)):

(2) $$\%\;\mathrm{Water}\;\mathrm{solubility}\;=\frac{w_{f}-w_{i}}{w_{f}}\times 100$$

where W_f_ and W_i_ are the films’ weight before (with no residual humidity, dried following the aforementioned conditions) and after the incubation into distilled water. All measurements were performed in three independent replicates.

#### 2.4.7. Water Vapor Transmission Rate

The water vapor transmission rate (WVTR) of each film (samples with 28 mm diameter) was assessed using test dishes and a chamber kept at 23 ± 2 °C with 53% relative humidity and an air velocity of *ca.* 160 m/min, as already described [7]. WVTR was calculated following Equation (3):

(3) $$\mathrm{WVTR}\;\left(\frac{g}{m^{2}}\times \;\mathrm{day}\right)=\frac{24\times \;X}{A\;\times \;Y}$$

where X refers to the weight gain expressed in grams, Y refers to the time in h for the weight gain of X, and A is the area exposed of each specimen (m^2^). Thee independent replicates were determined for each formulation.

#### 2.4.8. Statistical Analysis

The data from wettability, mechanical, chromatic, water solubility, and water vapor transmission rate were statistically evaluated by applying the Student’s *t*-test with a level of significant difference of 95% and *p* < 0.05.

## 3. Results and Discussion

Potato chip industry frying residues add a total of 42% (*w*/*w*) spent frying oil (SFO), mainly composed by oleic (50%), palmitic (33%), linoleic (12%), and stearic (3%) acid residues (Figure S1). The SFO peroxide value, indicative of oil oxidation promoted by thermal frying conditions [30], was 7.2 ± 0.5 milliequivalent of oxygen/kg of oil. Similar peroxide values have been reported for sunflower and soyabean oils (*ca.* 7.6 and 7.7 milliequivalent of oxygen/kg of oil, respectively) with 4 frying at 170–180 °C [27].

The influence of an alkaline catalyst (KOH) and SFO on the formulation of potato starch-based films was studied by measuring the films’ wettability, mechanical, chromatic, water solubility, and water vapor barrier properties.

### 3.1. Wettability of Potato Starch/SFO/KOH-Based Films

**Figure 1:**
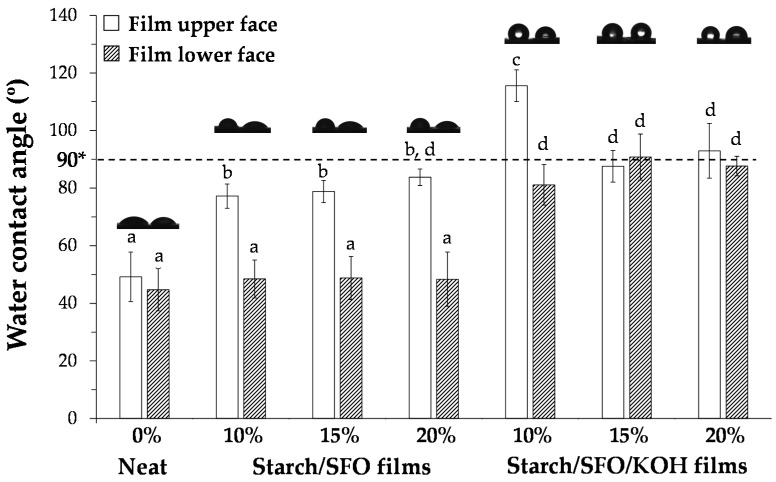
Water contact angles of “upper” and “lower” surfaces of potato starch-based films containing different SFO (spent frying oil) amounts (0, 10, 15 and 20% *w*/*w* related to starch dry weight) in the absence and presence of KOH (10% *w*/*w* related to the SFO weight). Different lowercase letters represent significantly different values (*p* < 0.05). * Hydrophobicity benchmark [31].

Figure 1 reveals the similarity of the water contact angles (WCAs) on both sides of the neat potato starch-based films (without SFO and KOH), highlighting their compositional homogeneity and hydrophilic character (*ca.* 50°). The incorporation of SFO significantly decreased the films hydrophilicity at the upper side (from *ca.* 50° to *ca.* 80°), keeping the WCA at *ca.* 50° in the films’ lower surface. This higher WCA variability between the starch/SFO-based films’ surfaces can be explained by the phase separation concerning water and SFO during casting and/or due to the micelles’ migration promoted by the different compounds’ density, similarly to what was observed in starch-based films when using waxes and oils recovered from potato industry byproducts [8]. These wettability profiles were similar for all the tested SFO loadings. In the presence of KOH, both upper and lower sides of the starch/SFO-based films became hydrophobic (*ca*. 90° on both sides), particularly when KOH and 15 and 20% SFO were used. Indeed, films containing KOH and 10% SFO still showed surfaces with heterogeneous wettability (*ca*. 115° at the upper and *ca.* 80° at the lower surface), showing the migration of lipidic components onto the films upper surface. The homogeneous wettability of the films containing the highest SFO concentrations suggests the starch modification with SFO-derived fatty acids through transesterification reactions.

### 3.2. Thickness and Mechanical Properties of Potato Starch/SFO/KOH-Based Films

**Figure 2:**
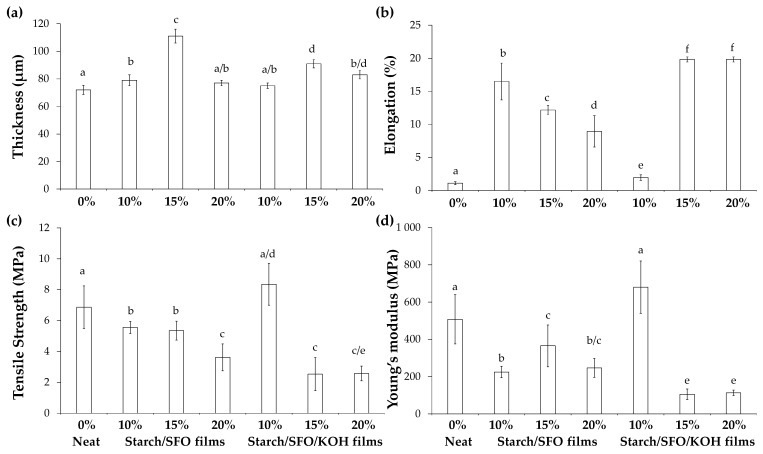
Thickness (**a**) and mechanical properties (elongation at break (**b**), tensile strength (**c**), and Young’s modulus (**d**) of potato starch-based films different SFO amounts (0, 10, 15 and 20% *w*/*w* related to starch dry weight) in the absence and presence of KOH (10% *w*/*w* related to the SFO weight). Different lowercase letters represent significantly different values (*p* < 0.05).

The neat potato starch-based films revealed a thickness of *ca.* 72 μm and a brittleness performance, reflected by their low tensile strength (*ca.* 7 MPa), elongation at break (*ca.* 1%), and high rigidity (Young’s modulus of *ca.* 507 MPa), following the tendency already reported [7,32]. Compared to the neat films, SFO incorporation increased the films’ elongation at break and decreased their tensile strength and Young’s modulus. Except for the 15% SFO amount, SFO had no significant influence in the films thickness. This amount of SFO seemed to promote the higher dispersion of starch and oil with a consequent maximum thickness. When the amount of oil increased to 20% an oil phase seems to be formed, explaining the decrease in the thickness of the films when compared to the 15% SFO. Moreover, the mechanical behavior was similar for all the SFO loadings tested (Figure 2), being more evident for the highest SFO amount used. The SFO presence in the starch matrix might have contributed to the decrease in the hydrogen cohesion forces within the amylose network, conferring a more flexible behavior to the starch/SFO-based films, similarly to what was observed when other lipidic compounds, such as potato waxes [8] and free fatty acids (palmitic, stearic, and oleic) were used [33]. Accordingly, the use of 1–3% oil in relation to starch (*w*/*w*) was shown not to be enough to change the starch-based films’ mechanical performance [8].

In the presence of KOH, although following the same mechanical trend as the films that only used SFO, the increase in the films’ elongation at break was even more evident when 15 and 20% SFO were used, as well as the decrease in the tensile strength and Young’s modulus. Therefore, for films containing 15 and 20% SFO, KOH increased by *ca.* 20× the stretchability and *ca.* 5× the elasticity of the neat materials, indicating that the starch might be modified with the SFO-derived fatty acids. The 10% SFO/KOH films presented an elongation, tensile strength, and Young’s Modulus values similar to the ones observed for the neat films. This can be explained by the modification of the starch to a certain extent, with an amount of free oil that was still high but not enough to change the mechanical performance of the starch-based films, when compared to the matrix containing a high amount of oil (15 and 20% SFO) that did not easily disperse in the aqueous starch medium. Thus, contrasting to starch/10% SFO/KOH-based films, 15 and 20% SFO amounts revealed to be in sufficient quantities to hinder the SFO-derived fatty acids dispersion, thus promoting the starch matrix discontinuity and decreasing the cohesion forces of the starch structure.

Based on wettability (Figure 1) and mechanical property results (Figure 2), the most hydrophobic, stretchable, and elastic properties were obtained when KOH and 15 and 20% SFO were incorporated into the starch-based formulations and no significant differences were observed among these two SFO amounts. With the aim of validating whether the starch network chemical modification was responsible for these changes, the structural (FTIR and ^1^H RMN assays), chromatic, water solubility, and water vapor barrier properties of starch-, starch/15% SFO-, and starch/15% SFO/KOH-based films were studied.

### 3.3. Structural Characterization of Potato Starch/15% SFO/KOH-Based Films

#### 3.3.1. FTIR Analysis

**Figure 3:**
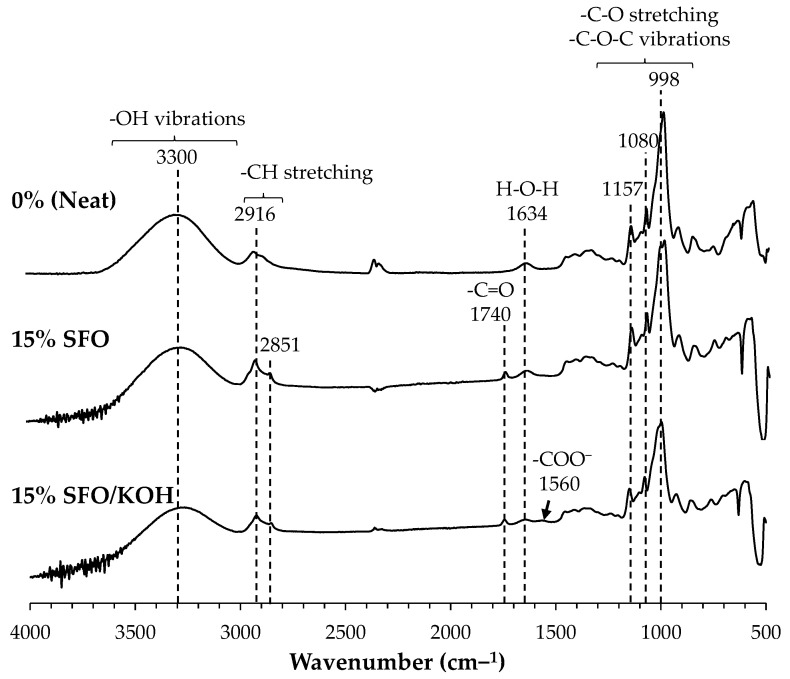
FTIR spectra of potato starch-based films containing 0% (neat), 15% SFO, and 15% SFO/KOH.

The FTIR profiles of the three developed formulations (Figure 3) present peaks at 1157 cm^−1^, 1080 cm^−1^, and 998 cm^−1^, which were characteristic of starch [34]. Besides, another peak at 1634 cm^−1^ appears, corresponding to the water molecules absorbed in the starch-based films. A strong and broad adsorption band at around 3000–3500 cm^−1^, attending its maximum at 3300 cm^−1^, was also observed, which is related to the hydroxyl groups of the potato starch. The FTIR spectra of both starch-based films with 15% SFO and 15% SFO/KOH showed a new band at 1740 cm^−1^ corresponding to the carbonyl group (C=O) that can be related, in the first case, to the fatty esters from the triacylglycerides of the SFO, or even, in the second case, from starch esters that can be formed in the presence of KOH [22]. Besides, the aliphatic region from the long SFO fatty acid chains is also clearly present at 2916 cm^−1^ and 2851 cm^−1^ in films containing 15% SFO and 15% SFO/KOH. Furthermore, only in the presence of KOH, a small peak at 1560 cm^−1^ was observed, corresponding to the carboxylate anion group (COO^−^) generated during the alkaline saponification of the SFO-derived fatty acids [35], suggesting that they can also be involved in the modification of the starch-based film properties.

#### 3.3.2. ^1^H NMR Analysis

**Figure 4:**
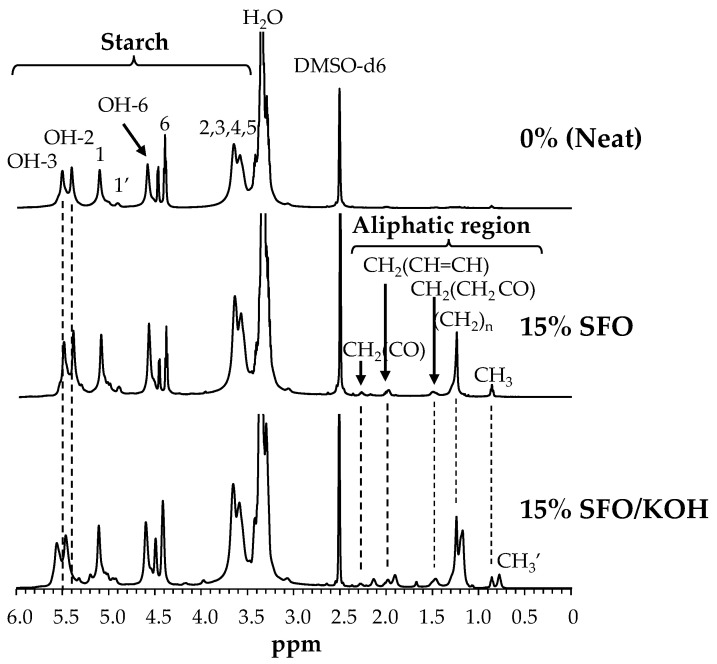
^1^H NMR spectra of potato starch-based films containing 0% (neat), 15% SFO, and 15% SFO/KOH, in DMSO-d6.

For the three developed formulations, the strongest signals were assigned as water (3.34 ppm) and DMSO-d6 (2.50 ppm) (Figure 4) [10]. In the ^1^H NMR spectrum of the neat film the starch characteristic proton resonance signals in the range of 3.50 ppm to 5.65 ppm were visible, which corresponded to the glucose ring protons. Particularly, the resonance signals at 5.40, 5.50 and 4.58 ppm were assigned to the 3 hydroxyl groups on C-2, C-3, and C-6 (OH-2, 3, and 6), respectively [15]. Moreover, at 5.10 ppm and 4.90 ppm it was possible to observe the signals of the anomeric hydrogen atom, denoted as H-1 and H-1′, corresponding to the starch α1,4 and α1,6 linkages signals, respectively [36].

For the ^1^H NMR spectrum of starch-based films with 15% SFO, the characteristic proton resonance bands of glucose units were still present, but new signals appeared at 0.87 ppm associated to the protons of the terminal methyl group (CH_3_), at 2.22 ppm associated to the methylene group (CH_2_) that occurs immediately before the carbonyl group of the ester, denoted as CH_2_(CO), which was linked to a second oxygen atom bonded to the carbon atom in the carbonyl group by a single bond. Other proton peaks at 1.24 ppm, 1.44 ppm, and 1.96 ppm, associated to other protons of different methylene groups of the SFO fatty acid chain [15], were also observed in films containing 15% SFO. However, in the presence of KOH, it was observed that the appearance of new spectral signals in the region of the aliphatic hydrogen atoms (CH_2_ groups) of the fatty acid chain (1.20 ppm, 1.67 ppm, 1.91 ppm, 2.12 ppm), as well as on the region of the terminal methyl groups at 0.76 ppm, denoted as CH_3_, were possible due to the chemical environment changes promoted by the starch modification in the presence of KOH. Furthermore, changes in the proton resonance of the OH-2 and OH-3 glucose units were observed (Figure 4), suggesting that the modification of the starch that occurred at the C-2 or C-3 positions, could have resulted from changed chemical environments due to the modification of the starch by the SFO-derived fatty acids in the presence of KOH. These changes may be due to the substitution of hydrogen atoms in the starch hydroxyl groups by the SFO-derived fatty acids.

### 3.4. Chromatic Properties of Potato Starch/15% SFO/KOH-Based Films

The L* values determined among the three tested film conditions were statistically similar (Table 1), revealing that, even in the presence of KOH, the incorporation of 15% SFO maintained the luminosity of the neat materials. However, both a* and b* values significantly changed. For starch-based films with 15% SFO and 15% SFO/KOH, the a* values decreased from 1.54 (neat film) to 1.27 and 0.89, respectively, while the b* values increased from −1.64 (neat film) to −0.99 and 0.23, respectively. Therefore, both SFO and SFO/KOH conferred a light yellowish coloration to the native films, this effect being more pronounced when KOH was included in the system. Moreover, the total color difference (ΔE) values varied from 0.75 to 1.98 for films with 15% SFO and 15% SFO/KOH, respectively. These differences may be related to the yellow-colored oxidized compounds that were present in the recovered SFO. These chromatic variations are in accordance to the bibliography, where the ΔE value was impacted by the incorporation of natural potato peel waxes [8] and beeswax, candelilla stalks, and carnauba leaves [37]. However, as can be observed in real images of the films in Table 1, the intrinsic films’ transparency was un-compromised by the usage of SFO and SFO/KOH.

### 3.5. Solubility and Water Vapor Barrier Properties of Potato Starch/15% SFO/KOH-Based Films

**Figure 5:**
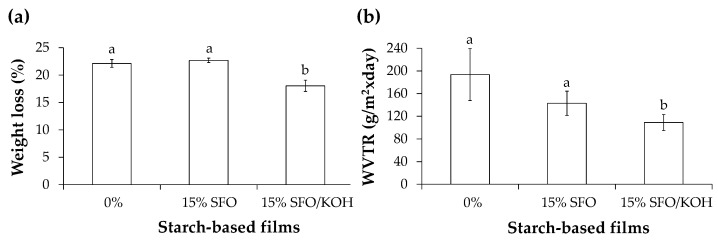
Weight loss (**a**) and water vapor transmission rate (WVTR) (**b**) of potato starch-based films containing 0%, 15% SFO, and 15% SFO/KOH. Different lowercase letters represent significantly different values (*p* < 0.05).

When in contact with aqueous environments, the neat films (0%) lost *ca.* 22% of their initial weight. A similar effect was observed for the starch/15% SFO-based films (*ca.* 23%). However, the presence of KOH minimized the weight loss (*ca.* 18%) of starch/15% SFO-based films, thus increasing their water tolerance (Figure 5a). This phenomenon supports the starch transesterification with SFO-derived fatty acids, decreasing the hydrogen bonding among the starch hydroxyl groups and the surrounding water molecules. As all starch-based films contained glycerol as plasticizing agent, the high-water solubility of this very hydrophilic molecule can explain the verified films’ weight loss after immersion into water [7].

The presence of KOH allowed the significant decrease of the WVTR of the starch-based films (Figure 5b). The incorporation of only 15% SFO during film production decreased the films’ WVTR, although did not show statistic difference when compared to the neat materials. Since the water vapor permeability occurred through the hydrophilic molecules that constituted the films [38], including amylose and amylopectin from potato starch, as well as glycerol, the highest WVTR decrease observed in the starch/15% SFO/KOH-based films reinforced the starch hydrophobization hypothesis by transesterification with the SFO fatty acids.

## 4. Conclusions

The incorporation of KOH during the starch/SFO-based films production revealed to be a promissory strategy for developing water tolerant biobased materials with enhanced stretchability and elasticity without changing their transparency, opening the possibility to extend the use of starch-based bioplastics to fields that include packaging applications. FTIR and ^1^H NMR suggested the possible modification of the starch structure by the presence of chemically bounded SFO-derived fatty acids. Therefore, this work establishes an in situ single-step approach for the simultaneous hydrophobization and plasticization of potato starch-based films, offering a new valorization strategy for the potato chip industry byproducts. The results obtained with the casting process used paves the way for the application of starch/SFO/KOH formulations by the plastic industries in their extrusion processes.

## Figures and Tables

**Table 1:** Real images and mean values of lightness (L*), red-green (a*), yellow-blue (b*), and total color difference (ΔE) of potato starch-based films containing 0% (neat), 15% SFO (spent frying oil), and 15% SFO/KOH. Different lowercase letters represent significantly different values (*p* < 0.05).

Starch-Based Films	Real Image	L*	a*	b*	ΔE
0% (neat)	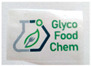	86.76 ± 0.42 ^a^	1.54 ± 0.04 ^a^	−1.64 ± 0.16 ^a^	-
15% SFO	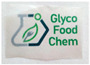	87.06 ± 0.32 ^a^	1.27 ± 0.07 ^b^	−0.99 ± 0.29 ^b^	0.75
15% SFO/KOH	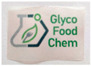	86.66 ± 0.35 ^a^	0.89 ± 0.07 ^c^	0.23 ± 0.31 ^c^	1.98

## Supplementary Materials

The following are available online at https://www.mdpi.com/article/10.3390/foods10122897/s1, Figure S1: Fatty acids composition (determined as FAME) of the spent frying oil recovered from potato chip industry frying residues.
