# Supplementary material for: Hydrophobic Starch-Based Films Using Potato Washing Slurries and Spent Frying Oil
Source: Foods. 2021 Nov 23;10(12):2897. doi: 10.3390/foods10122897 (PMC8700295; doi:10.3390/foods10122897)
Supplement: Supplementary file 1 [file foods-10-02897-s001.zip › foods-1439300-supplementary.pdf]

Hydrophobic starch-based films using potato washing slurries and spent frying oil

(Supplementary Figure)

Sílvia Petronilho*, André Oliveira, M. Rosário Domingues, Fernando M. Nunes, Manuel A. Coimbra, and Idalina Gonçalves,

* Correspondence: silviapetronilho@ua.pt

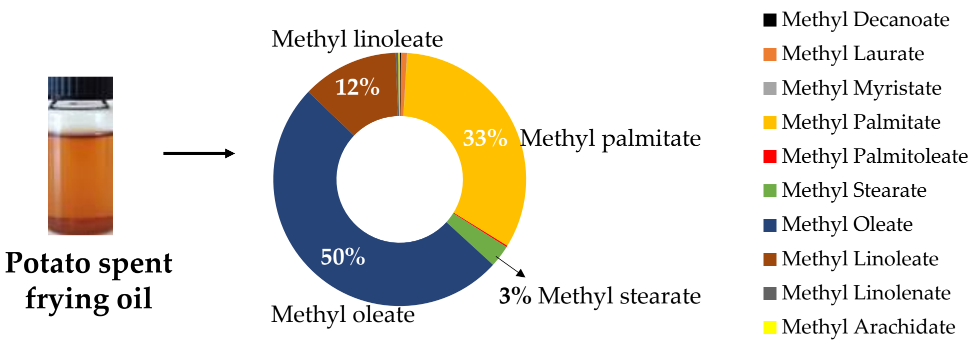

Figure S1. Fatty acids composition (determined as FAME) of the spent frying oil recovered from potato chips industry frying residues.
